# Large-Scale Plasma Proteomics and Genetic Integration Uncover Novel Biological Pathways in Male Pattern Baldness

**DOI:** 10.3390/ijms27042052

**Published:** 2026-02-22

**Authors:** Lingfeng Pan, Caihong Li, Philipp Moog, Samuel Knoedler, Haydar Kükrek, Ulf Dornseifer, Hans-Günther Machens, Jun Jiang

**Affiliations:** 1 Clinic for Plastic, Reconstructive and Hand Surgery, Klinikum Rechts der Isar, Technical University of Munich, 81675 Munich, Germany; 2 Department of Dermatology, University Hospital Erlangen, Friedrich-Alexander University of Erlangen-Nürnberg, 91054 Erlangen, Germany; 3 Department of Plastic, Reconstructive and Aesthetic Surgery, Isar Klinikum, 80331 Munich, Germany

**Keywords:** male pattern baldness, proteomics, genetics, therapeutic target, inflammaging

## Abstract

Male pattern baldness (MPB) is a highly prevalent condition with a complex, poorly understood molecular basis that limits therapeutic innovation. This study aimed to bridge the gap between statistical genetic associations and biological function by identifying and prioritizing causal proteins and pathways involved in MPB. Using data from 24,069 men in the UK Biobank, we performed a proteome-wide association study of 2911 plasma proteins with self-reported MPB severity via multivariable ordinal logistic regression, adjusting for age, Body Mass Index (BMI), ethnicity, lifestyle, socioeconomic factors, and testosterone levels. Significant proteins underwent pathway enrichment analysis. Genetic integration included MAGMA for gene-level aggregation and tissue prioritization, transcriptome-wide association studies (TWAS) with GTEx models, conditional fine-mapping, and validation in an independent scalp biopsy transcriptomics dataset (GSE90594). Druggability and pleiotropy were evaluated using databases and phenome-wide association studies. Forty-seven proteins were significantly associated with MPB severity, enriched in pathways involving epidermis development, hair cycle regulation, and cell adhesion. Multi-omic integration prioritized five independent candidate genes: *CD38*, *FGF5*, *TACSTD2*, *DPEP1*, and *PLB1*. Transcriptomic validation confirmed differential expression in balding scalp for *CD38* (upregulated) and *TACSTD2*, *PLB1* (downregulated). CD38 was identified as druggable with low pleiotropic risks. This study elucidates the molecular architecture of MPB, revealing novel biological pathways beyond canonical androgen signaling. By prioritizing promising non-hormonal targets like CD38, our findings provide a robust, evidence-based framework to guide the development of future therapeutic interventions for this common condition.

## 1. Introduction

Male pattern baldness (MPB), a highly prevalent condition affecting up to 80% of men by the age of 70 [1]. While often perceived as a cosmetic issue, it is associated with a significant psychosocial impact that can affect quality of life [2]. Currently available treatments include the oral 5α-reductase inhibitor finasteride, the topical vasodilator minoxidil, and surgical hair transplantation [3]. Furthermore, compelling epidemiological evidence has linked MPB to a higher risk of systemic diseases, including cardiovascular disease, prostate cancer, and metabolic syndrome, positioning it as a potential clinical marker for male health [4,5,6]. These epidemiological associations transform MPB from a skin-limited condition into a unique window into an individual’s broader cardiometabolic and tumor risk profile, necessitating a deeper understanding of the underlying molecular basis. However, the precise molecular pathways driving its progression remain incompletely elucidated, representing a key barrier to therapeutic development.

The hair follicle undergoes a continuous, cyclical process of growth consisting of three distinct phases: anagen (active growth), catagen (regression), and telogen (rest), followed by shedding of the hair shaft (exogen) and re-entry into anagen [7]. The duration of the anagen phase is the primary determinant of hair length and is tightly regulated by a complex interplay of signaling molecules within the follicular microenvironment [8]. The pathophysiology of MPB involves three key features: follicular miniaturization, alteration of the hair cycle, and perifollicular inflammation [9]. While this process is multifactorial, the principal androgen implicated in genetically susceptible individuals is dihydrotestosterone (DHT). DHT exerts its effects by binding to androgen receptors in the hair follicle, which not only shortens the anagen (growth) phase but may also prolong the telogen (resting) phase [10]. This interaction triggers complex signaling cascades, such as the Wnt/β-catenin and TGF-β pathways, ultimately leading to the progressive transformation of large, pigmented terminal hairs into fine, pale vellus hairs [11,12]. Furthermore, a low-grade, chronic inflammatory infiltrate is often observed around the follicles, potentially contributing to fibrosis and the irreversibility of the condition [13].

Following initial twin studies that estimated a heritability of up to 80% for MPB [14], genome-wide association studies (GWAS) have offered a more precise genetic inventory, identifying 63 risk loci that together account for approximately 39% of the phenotypic variance [15]. While these discoveries have been instrumental in confirming the complex genetic architecture of MPB, they primarily identified broad chromosomal regions of risk. The specific molecular intermediaries—particularly the proteins—that functionally connect these genetic variants to the pathophysiology of hair follicle miniaturization are still largely uncharacterized. This study therefore directly confronts a central challenge of the post-GWAS era: the systematic translation of statistical genetic associations into a functional understanding of disease biology and the identification of specific proteins that mediate genetic risk.

Proteomics offers a powerful approach to bridge this critical gap between genetics and pathophysiology by characterizing the functional molecular intermediaries. Nevertheless, previous studies in this field have often been limited by small sample sizes, insufficient control for critical confounding factors, or a narrow scope of protein analysis, thus failing to yield robust and replicable findings [16,17]. To overcome these limitations, we undertook a comprehensive, large-scale investigation using data from over 20,000 men in the UK Biobank. By integrating large-scale plasma proteomics with multivariable-adjusted regression, pathway analysis, and genetic methods such as transcriptome-wide association studies (TWAS), this study aims to provide a multi-omics framework to elucidate the molecular architecture of MPB and reveal targets for future intervention.

## 2. Results

### 2.1. Participant Characteristics

This study included a total of 24,069 male participants from the UK Biobank. The baseline demographic, lifestyle, and clinical characteristics of the cohort, stratified by four levels of MPB severity based on a validated pictogram-based self-assessment (Level 1: unaffected; Level 2: frontotemporal balding; Level 3: frontotemporal and vertex balding; Level 4: complete vertex baldness; Section 4.1), are presented in Table 1. The overall mean age of the cohort was 57.05 (SD 8.33) years, with a mean Body Mass Index (BMI) of 27.84 (SD 4.23) kg/m^2^. Statistically significant differences were observed across all measured baseline characteristics among the hair loss severity groups.

Specifically, the severity of hair loss demonstrated a clear positive association with both increasing age and BMI (both *p* < 0.001). Participants with the most severe hair loss (Level 4) were, on average, older (mean age 58.59 years) and had a higher BMI (mean 28.20 kg/m^2^) compared to those with the mildest hair loss, who had a mean age of 55.71 years and a mean BMI of 27.78 kg/m^2^. Similar trends were noted for lifestyle and socioeconomic factors. The proportion of previous smokers and individuals with a low level of education progressively increased with the severity of hair loss (both *p* < 0.001). While serum testosterone levels showed a statistically significant difference between the groups (*p* = 0.002), the absolute mean values exhibited only minimal variation, ranging from 11.86 nmol/L to 12.09 nmol/L.

### 2.2. Plasma Proteome-Wide Associations with Male Hair Loss Severity

**Figure 1:**
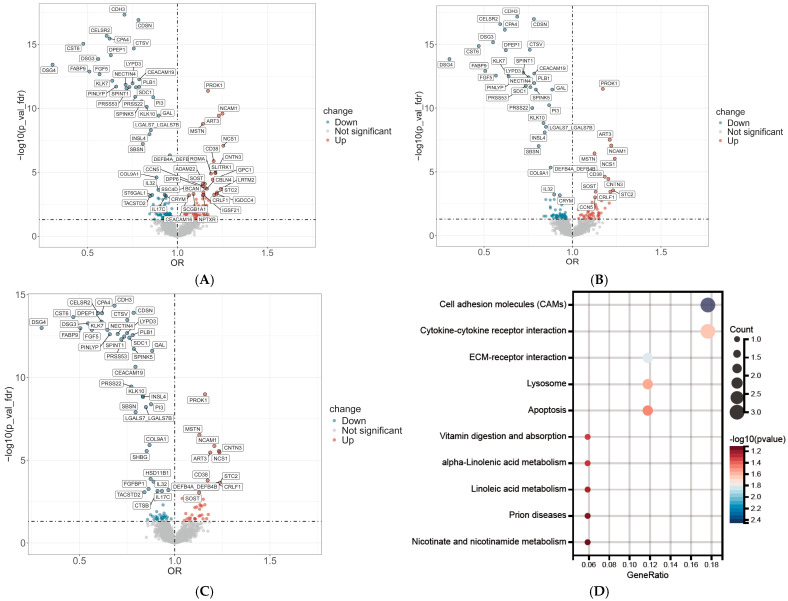

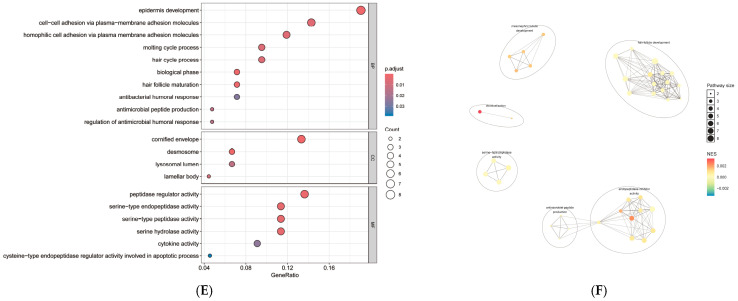
Proteome-wide association and functional enrichment analysis of proteins associated with male hair loss severity. (**A**) Volcano plots displaying the odds ratios (ORs) for the association between 2911 plasma proteins and the severity of hair loss. The horizontal dashed line represents the FDR significance threshold. Model 1, adjusted for age, ethnicity, and BMI, showing 60 significantly associated proteins. (**B**) Model 2, additionally adjusted for lifestyle and socioeconomic factors, showing 43 significant proteins. (**C**) The core set of 41 proteins that remained statistically significant across all three models (including Model 3, which also adjusted for testosterone). (**D**) Kyoto Encyclopedia of Genes and Genomes (KEGG) pathway enrichment analysis of the identified hair loss-associated proteins. (**E**) Gene Ontology (GO) enrichment analysis showing significantly enriched terms categorized by Biological Process, Cellular Component, and Molecular Function. (**F**) Functional network analysis visualizing the interconnected modules of enriched biological pathways, highlighting a large module central to hair cycle regulation and a secondary module related to antimicrobial peptide production. The color of the nodes corresponds to the Normalized Enrichment Score (NES).

In the proteome-wide association study involving 2911 plasma proteins, multiple proteins were identified as significantly associated with the severity of male hair loss after applying a stringent Bonferroni correction. In Model 1, which was adjusted for age, ethnicity, and BMI, 60 proteins showed significant associations with hair loss severity (Figure 1A). This number was reduced to 43 proteins in Model 2, which incorporated additional adjustments for lifestyle and socioeconomic factors (Figure 1B). After further adjustment for serum testosterone levels in Model 3, a total of 47 proteins remained significantly associated. Among these, a core set of 41 proteins exhibited highly robust associations, maintaining statistical significance across all three models (Figure 1C). The inclusion of serum testosterone in Model 3 did not weaken the overall number of associations but instead modified the set of significant proteins, increasing the count from 43 to 47. This suggests that testosterone may play a complex modulatory role beyond that of a simple confounder. Notably, four proteins—sex hormone-binding globulin (SHBG), fibroblast growth factor–binding protein 1 (FGFBP1), cathepsin B (CTSB), and 11-beta-hydroxysteroid dehydrogenase type 1 (HSD11B1)—emerged as significant only after adjustment for total testosterone levels. Detailed information on the relevant proteins in the models was provided in Supplementary Tables S1–S3.

### 2.3. Functional Characterization of Proteins Associated with Hair Loss Severity

To elucidate the underlying biological functions of the proteins associated with hair loss severity, we performed pathway and functional enrichment analyses. The associated proteins were significantly enriched in Kyoto Encyclopedia of Genes and Genomes (KEGG) pathways including ‘Cell adhesion molecules (CAMs)’, ‘Cytokine-cytokine receptor interaction’, and ‘ECM-receptor interaction’ (Figure 1D). Gene Ontology (GO) analysis provided a more granular view. For Biological Process, we observed significant enrichment in ‘epidermis development’, ‘hair cycle process’, and ‘cell–cell adhesion’. For Cellular Component, the proteins were primarily localized to the ‘cornified envelope’ and ‘desmosome’, while Molecular Function analysis highlighted an enrichment in ‘peptidase regulator activity’ (Figure 1E). These findings were synthesized through a functional network analysis, which visualized a large, interconnected module central to hair cycle regulation and a separate secondary module linking antimicrobial peptide production with endopeptidase inhibitor activity (Figure 1F). The consistently positive Normalized Enrichment Scores (NES) across the network indicate that these biological processes are systematically involved in the progression of hair loss severity.

### 2.4. Prioritization of MPB-Associated Tissues and Validation of Candidate Genes

**Figure 2:**
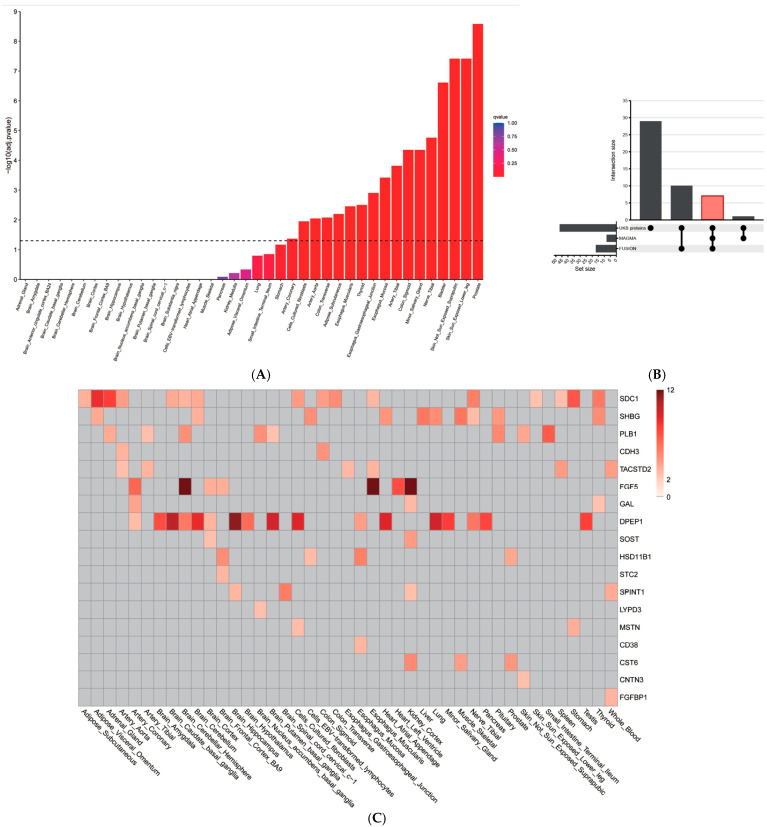
Genetic Validation and Tissue Prioritization of MPB-Associated Candidate Genes. (**A**) Results of the MAGMA gene-property analysis across 50 non-female-specific GTEx v8 tissues. The bar plot shows the enrichment of male pattern baldness (MPB) genetic associations within genes expressed in each tissue, with 35 tissues showing significant enrichment. (**B**) UpSet plot illustrating the overlap between the initial 47 candidate genes (from the UKB proteins), genes with significant gene-level associations (MAGMA), and genes with significant expression-level associations (FUSION). The central intersection highlights the seven genes (*CD38*, *TACSTD2*, *FGF5*, *DPEP1*, *PLB1*, *SDC1*, and *SHBG*) that are significant in both MAGMA and FUSION analyses. (**C**) Heatmap of the TWAS results from FUSION. This plot shows the association significance for genetically predicted gene expression and MPB risk, identifying 17 genes that surpassed the transcriptome-wide FDR < 0.05 threshold Red indicates statistically significant results (*p* < 0.05), and darker shades correspond to smaller *p*-values.

This study began with a set of 47 candidate genes, selected based on the significant association of their corresponding plasma proteins with MPB in the UK Biobank. To investigate the genetic mechanisms underlying these candidates, we first performed a gene-property analysis using MAGMA to identify the most relevant tissues. This analysis, which included 50 non-female-specific tissues from GTEx v8, revealed significant enrichment in 35 tissues (Figure 2A). The most significant enrichment signals fell into several broad categories, including male-reproductive tissues (e.g., Prostate, Testis), skin (e.g., Skin_Sun_Exposed_Lower_leg, Cells_Cultured_fibroblasts), the vascular system (e.g., Artery_Tibial, Artery_Aorta), and the nervous system (e.g., Nerve_Tibial). This suggests these tissues are primary sites where the genetic regulation of the candidate genes influences the pathophysiology of MPB.

To further validate these candidates, we next performed analyses at both the gene and transcript levels. First, gene-level association analysis using MAGMA confirmed that 8 genes were significantly associated with MPB (False Discovery Rate, FDR < 0.05; Supplementary Table S4). Second, a TWAS using FUSION identified 17 genes showing significant expression-level associations in the prioritized tissues (FDR < 0.05; Supplementary Table S5 and Figure 2C).

### 2.5. Integration of Analyses Identifies Core MPB Candidate Genes

To identify the most robust candidates supported by multiple lines of evidence, we integrated the results from the MAGMA and FUSION analyses. This comparison revealed a core set of 6 genes that were significantly associated with MPB at both the overall gene level (MAGMA) and the expression level (FUSION). These seven overlapping genes are *CD38*, *TACSTD2*, *FGF5*, *DPEP1*, *PLB1*, *SDC1* and *SHBG* (Table 2). This finding highlights a small group of high-confidence candidates, reinforcing their potential causal role in the pathogenesis of MPB. To further distinguish independent drivers within this group, we performed a conditional and joint (COJO) analysis. This fine-mapping step revealed that five of the genes—*CD38*, *TACSTD2*, *FGF5*, *DPEP1* and *PLB1*—remained significant, classifying them as jointly significant and representing robust, independent associations (indicated in green in Supplementary Figure S1). In contrast, the signal for *SDC1* and *SHBG* were attenuated and no longer significant, suggesting they are marginally significant gene whose associations were likely driven by LD with a primary signal (indicated in blue in Supplementary Figure S1). This integrated approach thus refines our findings to a high-confidence set of five genes with strong evidence for an independent role in the pathogenesis of MPB.

### 2.6. Validation of Prioritized Genes in Human Scalp Tissue

**Figure 3:**
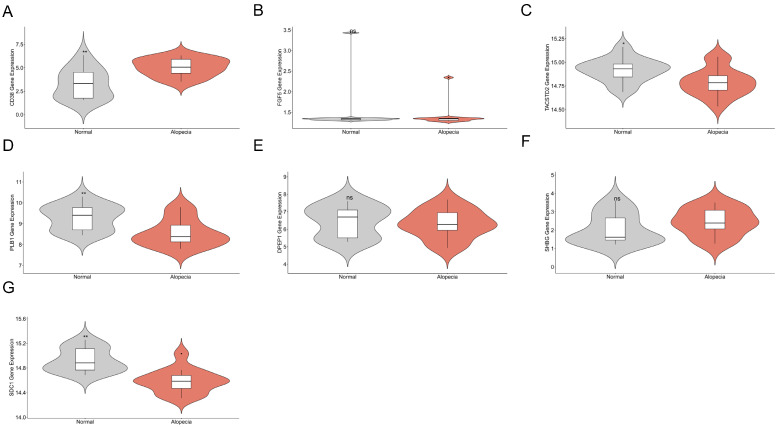
Transcriptomic Validation of Prioritized Genes in Human Scalp Tissue. Violin plots illustrating the differential expression of seven prioritized genes in scalp biopsies from the GSE90594 dataset, comparing 14 individuals with androgenetic alopecia (Alopecia) to 14 healthy controls (Normal). The plots correspond to the following genes: (**A**) CD38, (**B**) FGF5, (**C**) TACSTD2, (**D**) PLB1, (**E**) DPEP1, (**F**) SHBG, and (**G**) SDC1. * *p* < 0.05; ** *p* < 0.01; ns: not significant.

To corroborate our integrative analysis with direct evidence from diseased tissue, we investigated the expression of the prioritized genes in the GSE90594 scalp biopsy dataset [18]. The analysis revealed that *CD38* was significantly upregulated in androgenetic alopecia scalp tissue compared to controls (adjusted *p* < 0.05), while *PLB1*, *SDC1*, and *TACSTD2* were significantly downregulated (adjusted *p* < 0.05 for each) (Figure 3). No significant expression differences were observed for *DPEP1*, *FGF5*, and *SHBG*. The concordance between our predictions and the observed differential expression in relevant tissue provides strong orthogonal support, strengthens the credibility of our analysis, and highlights these candidates for functional follow-up.

### 2.7. Druggability and Pleiotropic Effects of Target Proteins

Among the proteins associated with MPB, only CD38 was identified as a target for existing drugs in the DGIdb database (Supplementary Table S6). To evaluate the potential for adverse pleiotropic effects from modulating CD38, a Phenome-Wide Association Study (PheWAS) was conducted. The analysis of a genetic proxy for plasma CD38 levels revealed no significant associations with any of the tested phenotypes after stringent correction for multiple testing (Supplementary Figure S2). These results suggest that therapeutically targeting *CD38* is unlikely to cause major off-target side effects.

## 3. Discussion

By integrating large-scale plasma proteomics with multi-layered genetic analyses in more than 20,000 men, this study tackled a central challenge of the post-GWAS era: translating statistical genetic associations into a functional understanding of disease biology. This comprehensive approach established a robust, evidence-based framework for prioritizing candidate drivers of MPB. The analysis began with a proteome-wide screen that identified 47 plasma proteins strongly associated with MPB severity. These candidates were systematically refined through gene-level, expression-level, and conditional genetic analyses, resulting in five prioritized independent candidate genes: *CD38*, *FGF5*, *TACSTD2*, *DPEP1*, and *PLB1*. This process provided a strong, evidence-based foundation for further biological investigation, moving beyond association to generate testable hypotheses about the molecular mechanisms underlying MPB.

Our results are highly consistent with the original transcriptomic analysis of GSE90594 dataset by Michel et al., who also reported significant upregulation of *CD38* and broad activation of inflammatory pathways. While the original study linked *CD38* primarily to immune cell recruitment, our multi-omic integration extends these findings by proposing *CD38*-mediated NAD^+^ depletion as a metabolic driver of follicular senescence (‘inflammaging’).

Our hypothesis for MPB integrates the established roles of androgen signaling and chronic inflammation with principles of metabolic aging by proposing a functional role for the ecto-enzyme CD38. CD38 is a multifunctional enzyme established as a primary consumer of nicotinamide adenine dinucleotide (NAD^+^) in aging tissues, and its upregulation is potently induced by pro-inflammatory signals secreted by senescent cells [19,20]. NAD^+^ is an essential coenzyme for cellular energy metabolism, and its age-related decline is a central hallmark of the aging process [21]. Based on these established findings, we hypothesize that, in genetically susceptible individuals, persistent androgen signaling and resulting chronic inflammation could induce cellular senescence in the follicular niche [22], leading to an inflammatory microenvironment whose SASP factors upregulate CD38 [23]. The subsequent depletion of the local NAD^+^ pool could trigger a bioenergetic crisis in the metabolically active cells of the anagen hair follicle, impairing their function and leading to progressive miniaturization [24]. This ‘localized inflammaging’ model reframes MPB as a potential process of premature biological aging within the scalp and suggests that the molecular machinery driving hair loss may overlap with that driving age-related dysfunction in other organs. Consequently, strategies aimed at inhibiting local CD38 activity or restoring the follicular NAD^+^ pool through precursor supplementation may represent novel approaches for mitigating the metabolic dysfunction underlying MPB [24,25].

Anti-CD38 monoclonal antibodies, including isatuximab and daratumumab, are FDA-approved for multiple myeloma, demonstrating that CD38 can be safely and effectively targeted in humans. This raises the possibility of developing strategies—potentially requiring localized delivery—to modulate its enzymatic activity in conditions of NAD^+^ depletion [26,27,28]. More accessible approaches involve natural CD38 inhibitors such as the flavonoids apigenin, quercetin, and luteolinidin, which show protective effects in preclinical models of metabolic and inflammatory diseases [29,30,31,32,33]. In parallel, experimental NAD^+^ boosting therapies using precursors such as NMN, NR, and vitamin B3 derivatives aim to restore depleted NAD^+^ pools by either inhibiting NAD-degrading enzymes or providing substrates for NAD^+^ synthesis [34,35,36]. To assess potential pleiotropic adverse effects, we performed a PheWAS using a genetic proxy for CD38 levels. As detailed in our results, the analysis revealed no significant associations with any tested phenotypes after stringent multiple-testing correction, suggesting that therapeutic targeting of CD38 is unlikely to cause major off-target effects.

Further supporting an inflammatory component, dipeptidase 1 (DPEP1) may act as a protective factor, consistent with its established role in modulating inflammatory pathways. DPEP1 is a zinc-dependent metalloproteinase that hydrolyzes and inactivates leukotriene D4 (LTD4), a potent pro-inflammatory lipid mediator [37]. This function is critical in various pathological settings; for example, DPEP1 has been shown to act as an adhesion receptor mediating neutrophil recruitment in models of lung and liver inflammation [38]. Its role is also well established in the kidney, where it contributes to inflammation and injury [39]. The protective signal for DPEP1 in our study is therefore notable, as its ability to regulate potent inflammatory mediators may be particularly relevant to the microinflammatory environment that contributes to hair follicle miniaturization in MPB.

The identification of well-established hair biology genes, including FGF5 and SHBG, provides strong internal validation of the multi-omic discovery pipeline. The convergence of this multi-stage analysis on known, biologically relevant genes further supports its ability to identify novel and equally valid candidates. FGF5 is a canonical negative regulator of the hair cycle and a key molecular switch that signals the termination of the anagen (growth) phase [40,41]. However, our findings reveal a more complex picture: FGF5 was associated with protection in plasma proteomics, risk in the TWAS, and no significant change in bulk scalp tissue analysis. These seemingly contradictory results likely reflect the complexity of its regulation. Because FGF5 is expressed locally in the outer root sheath during a narrow window at the end of the anagen phase, its signal may be diluted in bulk tissue analyses [42]. Moreover, systemic FGF5 levels may be regulated by feedback mechanisms that do not directly reflect its local follicular activity. The TWAS result, indicating that genetically predicted higher FGF5 expression is associated with MPB risk, aligns with its established function as a key initiator of the catagen (regression) phase. This suggests that the biological role of FGF5 may depend strongly on the timing and location of its expression.

This knowledge positions FGF5 inhibition as a promising strategy for treating hair loss. Although no pharmaceuticals targeting this pathway have been approved by regulatory agencies such as the FDA, alternative approaches have introduced FGF5-inhibiting products to consumers. Researchers have developed topical formulations containing botanical extracts with FGF5-inhibiting activity. These products have been evaluated in placebo-controlled clinical trials, which confirmed that their efficacy arises from prolonging the anagen phase rather than reactivating dormant follicles. One trial reported a significant improvement in the anagen-to-telogen ratio and a 44.2% increase in growing follicles after 16 weeks [43].

Unlike FGF5 and DPEP1, which act within local tissue environments, SHBG reflects systemic hormonal and metabolic status. As the primary transporter of androgens, SHBG shows an inverse correlation with bioactive free testosterone, explaining its strong protective association with MPB at the plasma protein level [44]. Beyond androgen binding, low SHBG levels are increasingly recognized as markers of insulin resistance and hyperglycemia, particularly in early-onset androgenetic alopecia [45]. This positions circulating SHBG as a robust systemic biomarker that integrates both hormonal and metabolic risk profiles. In contrast, genetic fine-mapping revealed only a marginal association with the *SHBG* gene, suggesting that its statistical signal likely reflects linkage disequilibrium with a neighboring causal gene. This distinction highlights that although plasma SHBG is a clinically informative risk indicator, common genetic variants regulating its systemic levels may not be direct causal drivers within the hair follicle—an important insight from integrating proteomic and genetic data.

Beyond canonical pathways, TACSTD2 and PLB1 implicate epithelial barrier dysfunction as a previously underappreciated contributor to MPB. TACSTD2 (Trop-2) is a transmembrane glycoprotein that maintains epithelial barrier integrity through interactions with tight junction proteins such as claudins [46]. Its marked downregulation in balding scalp suggests epithelial destabilization that may render follicular units more vulnerable to miniaturizing signals, consistent with the epithelial barrier hypothesis in chronic inflammatory skin diseases [47]. Concurrently, PLB1 encodes a phospholipase with dual A1/A2 activities expressed in human epidermis that promotes barrier function by converting lipids into free fatty acids. Reduced PLB1 expression may therefore disrupt the lipid composition required for follicular microenvironment integrity [48,49]. Together, these findings suggest that MPB progression involves impaired barrier function through both structural and lipid-mediated pathways, offering new insights beyond the conventional androgen-centric view.

This investigation is notable for its large scale, leveraging the UK Biobank cohort for robust statistical power, and for its innovative multi-omic integration, which establishes a coherent chain of evidence from systemic biomarkers to potential local drivers. Despite these strengths, several methodological limitations must be acknowledged. A primary limitation is the reliance on systemic (plasma) proteomics as a proxy for local (scalp) biology. The observed associations may reflect protein production in other tissues correlated with both MPB status and plasma levels. It remains unclear whether circulating proteins or those secreted locally by hair follicles are the primary mediators. Tissue-level proteomic studies are needed to confirm the local relevance of these findings. Another major challenge is the absence of a large-scale expression quantitative trait loci (eQTL) resource from human hair follicle tissue. Consequently, our TWAS analyses relied on data from other tissues, such as skin, which may not fully capture the genetic regulatory architecture of hair follicle cells. Additionally, the UK Biobank cohort is predominantly of European ancestry, which may limit the generalizability of our findings. The prevalence, genetic architecture, and environmental modifiers of MPB are known to vary across ethnic populations. Replication of this multi-omic framework in geographically and ethnically diverse cohorts will be important to determine whether the identified associations are consistent or whether population-specific signals emerge. Furthermore, psychological factors such as stress and depression, which have been implicated in hair loss, were not incorporated as covariates in the present analysis. The complex bidirectional relationship between psychological conditions and MPB warrants a dedicated investigation with an appropriate analytical framework, and represents an important direction for future research.

Validation in the GSE90594 scalp biopsy dataset provided supporting evidence for several candidates, but its modest sample size and reliance on bulk RNA sequencing impose constraints. Bulk RNA analysis averages gene expression across cell types, potentially obscuring signals from rare but critical populations such as stem cells, dermal papilla cells, or immune infiltrates. To overcome these limitations and extend our hypotheses, single-cell and spatial transcriptomics should be applied to scalp biopsies from individuals at different stages of MPB. Recent studies demonstrate that these technologies can resolve the heterogeneous landscape of hair follicles in androgenetic alopecia, identifying key cell populations and pathways in disease progression. Applying these approaches would allow candidate genes to be mapped to specific cell types, providing a high-resolution view of their roles in follicular miniaturization and advancing targeted insights into MPB.

## 4. Materials and Methods

**Figure 4:**
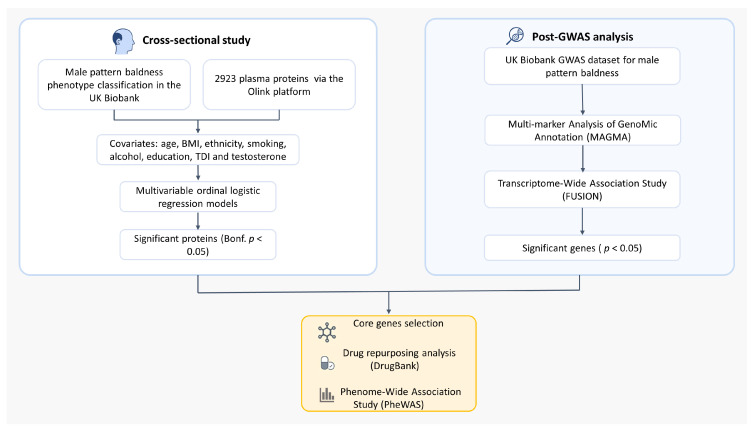
Multi-omic study design integrating proteome-wide association analysis with a post-GWAS pipeline to identify and prioritize candidate genes for male pattern baldness.

This study integrates a cross-sectional proteome-wide association study in the UK Biobank with a post-GWAS analysis (Figure 4). We first identified proteins associated with MPB, then used genetic data (MAGMA, TWAS) to prioritize genes. By integrating these multi-omic results, we selected a core set of candidate genes for transcriptomic validation, druggability assessment, and safety evaluation via a PheWAS.

### 4.1. Study Population

This study utilized data from the UK Biobank (UKB), a large-scale, long-term prospective cohort study that recruited over 500,000 participants aged 40–69 across the United Kingdom between 2006 and 2010. All participants provided written informed consent, and the UK Biobank has approval from the North West Multi-centre Research Ethics Committee. This research was conducted under UK Biobank application number [687300].

Participants for the hair loss analysis were drawn from the male cohort of the UK Biobank. This analysis utilizes data from the initial assessment visit between 2006 and 2010, where participants were recruited and gave consent. Using a touch-screen questionnaire, participants self-reported their degree of MPB by selecting one of four pictograms that best matched their hair pattern. The scores were defined as follows: 1—Unaffected; 2—Frontotemporal balding; 3—Balding of the frontotemporal region and vertex; and 4—Complete baldness of the top of the scalp. The initial assessment included 226,801 male participants, making this the largest investigation of self-reported MPB to date. For this study, individuals with incomplete phenotypic data for this measure were excluded.

### 4.2. Plasma Proteomic Profiling

Plasma protein measurements were obtained from the UK Biobank Pharma Proteomics Project (UKB-PPP), which profiled approximately 54,000 participants. Baseline blood samples, collected between 2006 and 2010, were stored in EDTA tubes at −80 °C prior to analysis. The proteomic profiling was conducted using the Olink^®^ Explore 3072 platform, measuring 2923 unique proteins across eight panels (cardiometabolic, cardiometabolic II, inflammation, inflammation II, neurology, neurology II, oncology, and oncology II). Protein expression levels were quantified as Normalized Protein eXpression (NPX) values on a log2 scale. Following extensive quality control, proteins with missing values in >20% of participants were excluded. All protein levels were standardized before being used in subsequent analyses. Finally, 2911 proteins were enrolled and investigated in this study.

### 4.3. Covariate Assessment

A comprehensive set of covariates was collected at the baseline assessment visit through touchscreen questionnaires, verbal interviews, and physical measurements. These included age and BMI, which were treated as continuous variables based on their measured values. Lifestyle factors included smoking status (categorized as ‘never’, ‘former’, or ‘current’). Total alcohol consumption was calculated based on self-reported weekly or monthly intake of various beverages. Standard units of alcohol were assigned as follows: 2 units (16 g) per pint of beer/cider, 1.5 units (12 g) per glass of wine or other alcoholic beverages, and 1 unit (8 g) per measure of spirits. Socioeconomic factors included educational attainment, which was classified into three groups: high (college or university degree), moderate (A-levels, AS-levels, O-levels/GCSEs, NVQ/HND/HNC, or other professional qualifications), and low (none of the above), and the Townsend Deprivation Index (TDI), a composite area-level measure of socioeconomic deprivation derived from census data on unemployment, non-car ownership, non-home ownership, and household overcrowding, with higher values indicating greater deprivation. Additionally, serum testosterone levels, obtained from biochemical assays, were included as a standardized continuous variable.

For the baseline characteristics comparison across the four hair loss groups, statistical tests were chosen based on the data distribution. Continuous variables following a normal distribution (e.g., age, BMI, testosterone) were presented as mean (standard deviation) and compared using one-way Analysis of Variance (ANOVA). Continuous variables with a skewed distribution (e.g., alcohol units, Townsend Deprivation Index) were presented as median (interquartile range) and compared using the Kruskal–Wallis test. Categorical variables (e.g., smoking status, education level) were presented as counts (percentages) and compared using the Chi-squared (χ^2^) test. A *p*-value of <0.05 was considered statistically significant.

### 4.4. Statistical Analysis

To investigate the association between 2911 standardized plasma protein levels and the severity of hair loss, treated as an ordinal variable (from 1 = no hair loss to 4 = severe hair loss), we performed a series of multivariable ordinal logistic regression analyses to calculate Odds Ratios (ORs) and their corresponding 95% Confidence Intervals (CIs). We employed three sequentially adjusted models to control for potential confounding: Model 1 adjusted for age, ethnicity, and BMI; Model 2 further adjusted for smoking status, alcohol consumption, educational attainment, and the Townsend Deprivation Index; Model 3 included all covariates from Model 2 with additional adjustment for serum testosterone level. To account for multiple testing across all proteins, the primary threshold for statistical significance was set using the stringent Bonferroni correction. Additionally, we applied the Benjamini–Hochberg procedure to control the FDR at 5% in exploratory analyses to identify potential associations warranting further investigation.

### 4.5. Pathway Enrichment

To explore the underlying biological mechanisms of the identified hair loss-associated proteins, we performed pathway enrichment analysis using the R package clusterProfiler (version 4.10.0), utilizing gene set databases encompassing GO terms (Biological Process (BP), Cellular Component (CC), and Molecular Function (MF)) and the KEGG. Faced with the challenge of interpreting the resulting large and often redundant list of pathways, we further employed the R package aPEAR (Advanced Pathway Enrichment Analysis Representation) (version 1.2.0). This tool aids in interpretation by leveraging gene-set similarities to group redundant pathways into interconnected clusters. aPEAR then assigns a biologically meaningful name to each cluster and visualizes the results as a network, enabling a more objective and automated overview of the key biological themes.

### 4.6. GWAS Sources for MPB

We utilized publicly available GWAS summary statistics for MPB from a study by Yap et al. [50]. This large-scale GWAS was conducted on 205,327 males of European ancestry from the UK Biobank. The analysis included a total of 18,065,087 autosomal and 1,064,602 X-chromosome single nucleotide polymorphisms (SNPs). In the original study, the MPB phenotype was adjusted for age, assessment center, ethnicity, and the first 40 genetic principal components to account for population structure. For complete details on the quality control procedures and analysis methods, please refer to the original publication by Yap et al.

### 4.7. Gene-Level Aggregation and Tissue Prioritization Using MAGMA

To convert SNP-level associations to gene-level signals, we performed a gene-based association analysis using MAGMA (version 1.08), a tool that accounts for linkage disequilibrium (LD) between SNPs [51]. SNPs from the MPB GWAS were mapped to protein-coding genes (NCBI Build 37), with a 5 kb upstream and 1.5 kb downstream window around each gene. We used the European panel from the 1000 Genomes Project Phase 3 as the LD reference panel to model the SNP correlation structure [52]. An FDR method was used to correct for multiple testing across all tested genes. As the GWAS cohort consisted entirely of males, four female-specific tissues (Vagina, Uterus, Ovary, and Fallopian Tube) were excluded from the Genotype-Tissue Expression (GTEx) v8 project’s list of tissues. This analysis tested, for each of the remaining 50 tissues individually, whether tissue-specific gene expression levels were predictive of MPB-related genetic associations. The analysis controlled for potential confounders such as gene size and SNP density. An FDR correction was applied to the results from the 50 tissue-specific analyses, and tissues that passed this significance threshold were selected for further TWAS analysis.

### 4.8. Transcriptome-Wide Association Study Using FUSION

For the TWAS, we employed the FUSION software (version 3.0) to identify genes whose genetically predicted expression is associated with MPB. We utilized pre-computed gene expression prediction models from the GTEx v8 project for the tissues prioritized in the MAGMA analysis. These models were built using SNPs within a 1 Mb window of each gene’s transcription start and end sites, with five regularized linear models (Best Linear Unbiased Prediction (BLUP), Bayesian Sparse Linear Mixed Model (BSLMM), LASSO, Elastic Net, and the top cis-eQTL) being evaluated for each gene-tissue pair [53]. The model with the best cross-validation performance, as determined by R^2^, was selected for each gene-tissue pair. The association test was performed using the MPB GWAS summary statistics and the 1000 Genomes EUR panel as the LD reference, with a transcriptome-wide FDR < 0.05 used to define significant associations.

### 4.9. Fine-Mapping of Independent Signals

To identify independent genetic signals, we conducted a COJO analysis using FUSION, which fits a joint model including all significant genes in a locus to estimate their conditional effects. Genes that remained significant after conditioning were classified as jointly significant (independent signals), while those whose associations were attenuated were classified as marginally significant [54].

### 4.10. Transcriptomic Validation in an Independent Dataset

Given the lack of available eQTL (expression quantitative trait loci) data from human hair follicle tissue, we sought to provide direct histological evidence for genes prioritized by our plasma protein and TWAS analyses. To achieve this, we validated their differential expression using the public transcriptomic dataset GSE90594, deposited in the NCBI Gene Expression Omnibus (GEO; https://www.ncbi.nlm.nih.gov/geo/, accessed on 21 February 2026). This dataset was generated by Michel et al. [18]. This dataset comprises vertex scalp biopsies from 14 males with androgenetic alopecia and 14 healthy male controls. A targeted differential expression analysis was performed on the log2-transformed expression values using the limma package in R. *p*-values were adjusted for multiple comparisons using the Benjamini–Hochberg method, with a significance threshold of <0.05. Results were visualized as violin plots using the ggplot2 package.

### 4.11. Druggability and Safety Assessment

The druggability of hair loss-associated proteins was systematically evaluated using the Drug-Gene Interaction Database (DGIdb), the Open Targets database, and the Therapeutic Target Database. We queried the database to identify established drug-gene interactions for our proteins of interest. To assess the potential for pleiotropic side effects and evaluate therapeutic safety, a subsequent PheWAS was conducted using summary statistics from AstraZeneca’s PheWAS Portal (https://azphewas.com/). This gene-based analysis utilizes genetic variants strongly associated with the target gene’s expression (primarily cis-expression Quantitative Trait Loci, or cis-eQTLs) as genetic instruments to investigate the phenome-wide consequences of the cumulative effects of genetically predicted expression of each target gene. This analysis leveraged comprehensive genetic and phenotypic information from approximately 450,000 participants in the UK Biobank, encompassing a wide range of both binary and continuous traits. A genome-wide significance threshold of *p* < 1 × 10^−8^ was applied to identify any potential off-target effects.

## 5. Conclusions

This comprehensive multi-omic study, integrating plasma proteomics with advanced genetic analyses in more than 20,000 men from the UK Biobank, elucidates the molecular architecture of MPB. Refining proteome-wide associations through gene-level, transcriptome-wide, and conditional analyses, we prioritized five independent candidate genes—CD38, FGF5, TACSTD2, DPEP1, and PLB1—thereby validating established regulators and uncovering novel roles in epithelial integrity, lipid metabolism, and inflammaging. CD38 implicates a testable hypothesis linking chronic inflammation, cellular senescence, and NAD^+^ depletion to follicular miniaturization. These insights advance understanding of MPB pathophysiology and highlight promising non-hormonal therapeutic targets for future interventions.

## Figures and Tables

**Table 1:** Baseline characteristics of the population with male pattern baldness.

Characteristics	Overall (n = 24,069)	Level 1 (n = 7848)	Level 2 (n = 5448)	Level 3 (n = 6382)	Level 4 (n = 4391)	*p*-Value
Age, years, mean (SD)	57.05 (8.33)	55.71 (8.55)	55.57 (8.56)	58.91 (7.64)	58.59 (7.77)	<0.001
BMI, kg/m^2^, mean (SD)	27.84 (4.23)	27.78 (4.33)	27.59 (4.12)	27.89 (4.20)	28.20 (4.20)	<0.001
Ethnicity, n (%)						
Other	2168 (9.0)	830 (10.6)	421 (7.7)	518 (8.1)	399 (9.1)	<0.001
White	21,901 (91.0)	7018 (89.4)	5027 (92.3)	5864 (91.9)	3992 (90.9)	
Smoking status, n (%)						
Current	3003 (12.5)	1108 (14.1)	693 (12.7)	768 (12.0)	434 (9.9)	<0.001
Never	11,591 (48.2)	3854 (49.1)	2601 (47.7)	3015 (47.2)	2121 (48.3)	
Prefer not to answer	78 (0.3)	19 (0.2)	22 (0.4)	24 (0.4)	13 (0.3)	
Previous	9397 (39.0)	2867 (36.5)	2132 (39.1)	2575 (40.3)	1823 (41.5)	
Alcohol intake, units/month, median [IQR]	13.00 [3.50, 25.00]	13.00 [3.50, 26.00]	14.00 [4.00, 25.00]	13.00 [4.00, 25.00]	12.00 [3.00, 24.50]	0.012
Education, n (%)						
low	4578 (19.0)	1388 (17.7)	959 (17.6)	1285 (20.1)	946 (21.5)	<0.001
moderate	11,364 (47.2)	3676 (46.8)	2646 (48.6)	2991 (46.9)	2051 (46.7)	
high	8127 (33.8)	2784 (35.5)	1843 (33.8)	2106 (33.0)	1394 (31.7)	
Townsend Deprivation Index, median [IQR]	−2.07 [−3.64, 0.84]	−1.94 [−3.63, 1.07]	−2.01 [−3.60, 0.73]	−2.24 [−3.69, 0.67]	−2.08 [−3.63, 0.80]	<0.001
Testosterone, nmol/L, mean (SD)	11.99 (3.71)	11.86 (3.72)	12.09 (3.77)	11.99 (3.66)	12.09 (3.69)	0.002

BMI, body mass index; SD, standard deviation; IQR, interquartile range. The Townsend Deprivation Index is a composite area-level measure of socioeconomic deprivation, with higher (less negative) values indicating greater deprivation.

**Table 2:** Multi-omic evidence summary for high-confidence candidate genes in male pattern baldness.

Gene Symbol	Name	Proteomic Association (Model 3 OR [95% CI])	Gene-Level Genetic Evidence (MAGMA ZSTAT)	Expression-Level Genetic Evidence (TWAS.Z & Top Tissue)	Scalp Tissue Validation (GSE90594 Direction)	Causal Inference Status (COJO Result)
CD38	cyclic ADP ribose hydrolase	1.17 [1.10–1.25]	3	3.23	Upregulated	Independent
FGF5	Fibroblast growth factor 5	0.57 [0.53–0.60]	7.21	5.02	No significant change	Independent
TACSTD2	Tumor-associated calcium signal transducer 2	0.84 [0.78–0.91]	2.98	−3.37	Downregulated	Independent
PLB1	Phospholipase B1	0.78 [0.75–0.81]	2.57	−3.26	Downregulated	Independent
DPEP1	Dipeptidase 1	0.62 [0.59–0.65]	6.56	−3.85	No significant change	Independent
SHBG	Sex hormone-binding globulin	0.85 [0.81–0.90]	3.35	−3.05	No significant change	Marginal
SDC1	Syndecan-1	0.76 [0.72–0.80]	6.1	−2.92	Downregulated	Marginal

OR, odds ratio; CI, confidence interval; MAGMA, Multi-marker Analysis of GenoMic Annotation; TWAS, transcriptome-wide association study; COJO, conditional and joint analysis.

## Data Availability

The data used in this study were obtained from the UK Biobank under application number [687300]. The datasets presented in this article are not readily available because of the UK Biobank data access and sharing restrictions. Requests to access the datasets should be directed to UK Biobank.

## Supplementary Materials

The following supporting information can be downloaded at: https://www.mdpi.com/article/10.3390/ijms27042052/s1.
