# Supplementary material for: Large-Scale Plasma Proteomics and Genetic Integration Uncover Novel Biological Pathways in Male Pattern Baldness
Source: Int J Mol Sci. 2026 Feb 22;27(4):2052. doi: 10.3390/ijms27042052 (PMC12940227; doi:10.3390/ijms27042052)
Supplement: Supplementary file 1 [file ijms-27-02052-s001.zip › Supplementary Figures.pdf]

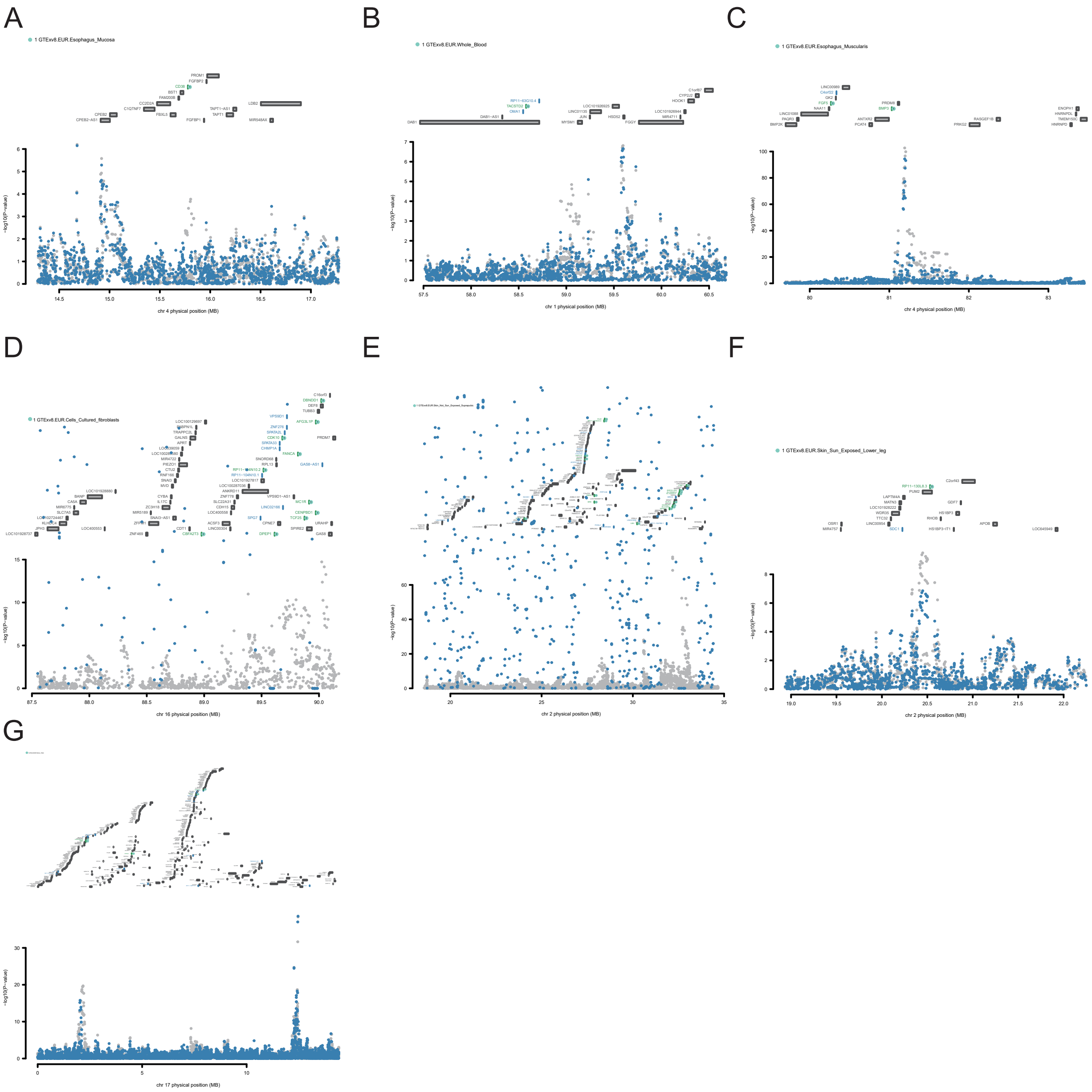

Supplementary Figure 1: Regional Association Plots of Loci with Significant Genes Identified by TWAS and Fine-Mapped by COJO.

The plots display the results of the Transcriptome-Wide Association Study (TWAS) for Male Pattern Baldness (MPB) at seven significant gene loci. The y-axis shows the -log10(P-value) for gene-based association, while the x-axis indicates the chromosomal position. Gene models are shown at the top of each plot. The highlighted genes were further analyzed by conditional and joint (COJO) analysis to distinguish independent signals. Genes determined to be jointly significant (independent signals) are highlighted in green. Genes whose association was attenuated and classified as marginally significant are highlighted in blue.The panels correspond to the following genes:(A) CD38 (jointly significant); (B) TACSTD2 (jointly significant); (C) FGF5 (jointly significant); (D) DPEP1 (jointly significant); (E) PLB (jointly significant); (F) SDC1 (marginally significant);(G) SHBG (marginally significant)

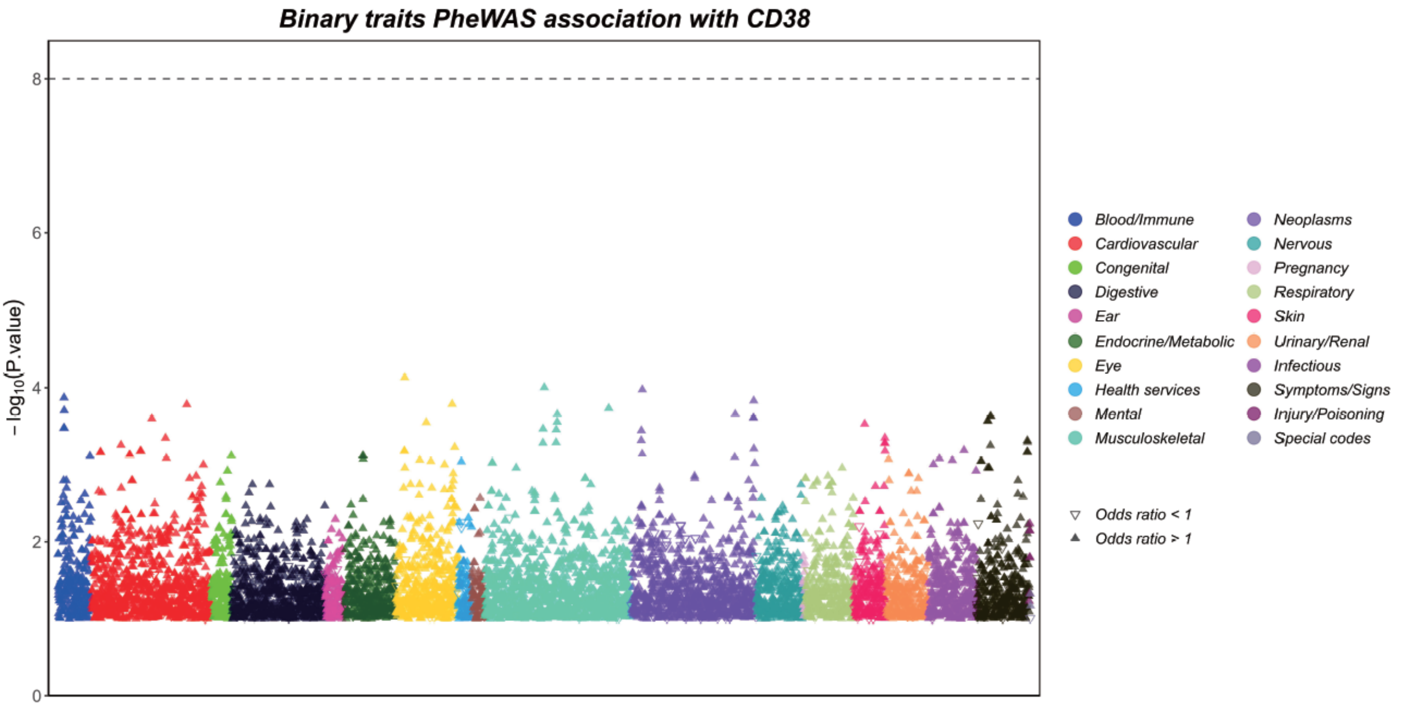

(a)

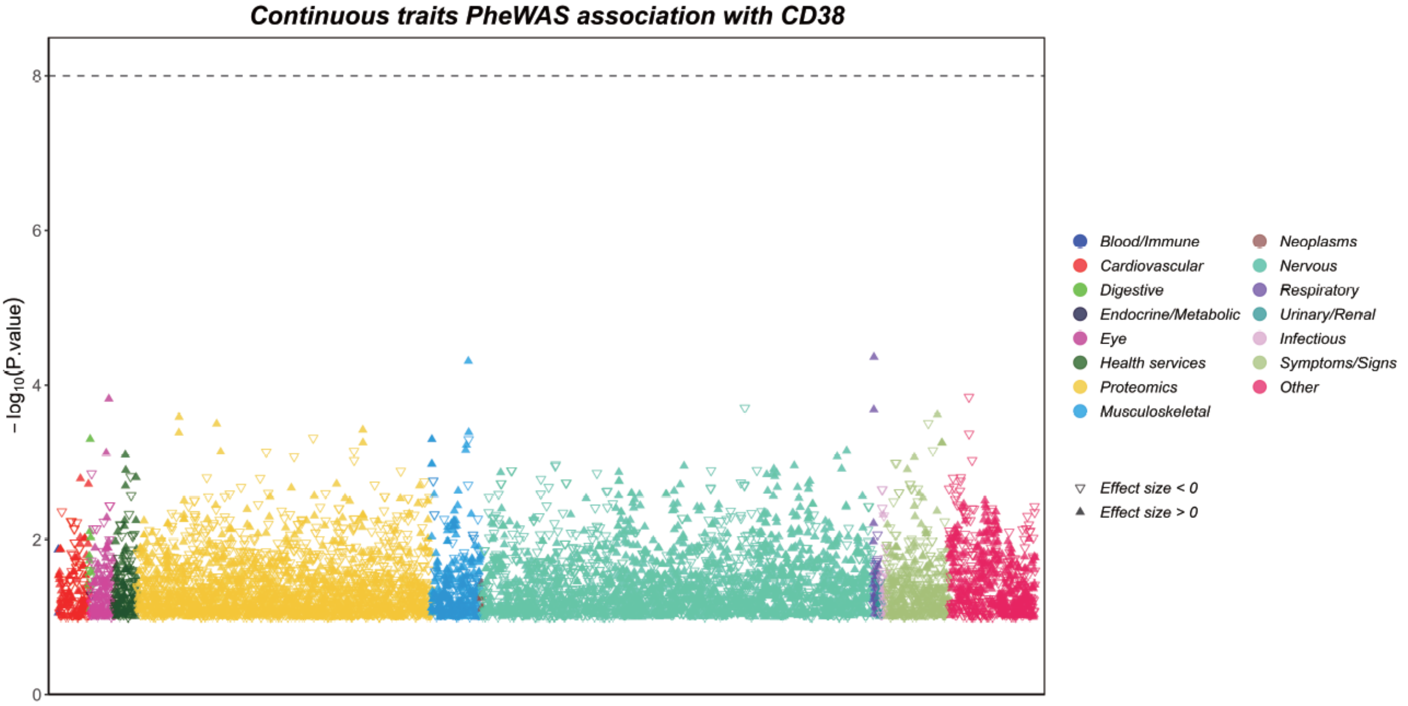

(b)

Supplementary Figure 2. Phenome-Wide Association Study (PheWAS) of Genetically Predicted CD38 Levels. Manhattan plots illustrating the association between a genetic proxy for plasma CD38 levels and a comprehensive range of phenotypes from the UK Biobank, conducted to assess potential pleiotropic effects. The analysis covers (A) binary traits and (B) continuous traits. Each point represents a distinct phenotype, categorized by physiological system on the x-axis. The horizontal dashed line indicates the genome-wide significance threshold (P = 1 × 10^{-8}). The analysis suggests a low risk of major off-target side effects, as the genetic instrument for plasma CD38 levels showed no significant association with any phenotypes other than plasma CD38 protein expression itself after stringent multiple testing correction.
